# Rapid and label-free identification of single foodborne pathogens using microfluidic pore sensors

**DOI:** 10.3389/fnut.2022.959317

**Published:** 2022-08-04

**Authors:** Tao Yang, Zisheng Luo, Ricardo A. Wu, Li Li, Yanqun Xu, Tian Ding, Xingyu Lin

**Affiliations:** ^1^State Key Laboratory of Fluid Power and Mechatronic Systems, College of Biosystems Engineering and Food Science, Fuli Institute of Food Science, Zhejiang University, Hangzhou, China; ^2^Key Laboratory of Agro-Products Postharvest Handling of Ministry of Agriculture and Rural Affairs, Zhejiang University, Hangzhou, China; ^3^Ningbo Research Institute, Zhejiang University, Ningbo, China

**Keywords:** microfluidics, solid-state micropore, foodborne pathogen, bacterial identification, label-free

## Abstract

Foodborne pathogenic microorganisms have become major threats that endanger human life and health. The current technology cannot perform rapid screening of foodborne pathogenic bacteria, and fail to timely control food safety risks. Here, we develop a novel microfluidic sensor for real-time and label-free bacteria classification at the single-cell level. Concretely, a low-aspect-ratio SiN micropore with PDMS coating was fabricated, which could significantly reduce the noise of the sensing system, and makes the microfluidic pore sensor sensitive to bacteria discrimination. The prepared SiN micropore equipped with the high temporal-spatial resolution was applied to observe bacterial translocation “events” and the current pulse signals could be obtained, which depend on the size, charge, and morphology of the target bacteria. According to the variation of the current pulse signals produced by different bacteria across the micropore, three common foodborne pathogens such as *Salmonella enteric, Listeria monocytogenes*, and *Escherichia coli* were identified. Due to convenience, rapidity, and accuracy, the label-free method we report here has great potential for the identification of diverse foodborne microorganisms at single-cell sensitivity.

## Introduction

Foodborne diseases have long been a serious threat to public health all over the world, and pathogenic bacteria are the main cause of these diseases (1). Foodborne pathogens include *Salmonella, Listeria monocytogenes, Staphylococcus aureus, Vibrio parahaemolyticus, Escherichia coli*, etc. These pathogenic bacteria can cause diarrhea, vomiting, fever, septicemia, and even death. It is estimated that there are 37 million illnesses caused by the foodborne pathogenic microorganisms in the world each year, with 230 thousand hospitalizations and 2.6 thousand deaths (CDC). Once the food is potentially contaminated, the bacteria may proliferate in large numbers in a short period of time, which may cause serious harm to human health. Therefore, it is important to determine the bacterial species timely and accurately, which helps to control risk and ensure food safety (2, 3).

Up to now, several available methods and techniques for bacterial identification have been developed, namely, API Bacteria Test System (4), DNA sequencing (5), 16S ribosomal RNA-based sequencing (6), immunological methods (7), and pulsed-field gel electrophoresis (8). These methods are generally stable and accurate. However, they are time-consuming, labor-intensive, and inherently rely on costly instruments and time-consuming treatments. With more researches focused on single cell analysis, how to realize a label-free, non-invasive, and real-time method for single foodborne bacterium identification has become a hot topic, but remain challenging.

In recent years, microfluidic technology has developed rapidly, and many microfluidic chips have been developed and applied (9–12). Microfluidic-based nano/micropore analysis technology is an emerging single-molecule analysis method that has been widely used in DNA sequencing (13, 14), protein sequencing (15), molecular recognition (16, 17), and virus detection (18, 19). Their sensing principle is based on the Coulter principle that detects of temporal changes in ion transport across the pore. Unlike Coulter counters, the sensing area of the chip is only nanometer-thick in thickness, which significantly increases the importance of the ionic resistance of the electrolyte solution outside the pore channel, thus enabling fast kinetic processes with ultra-high temporal-spatial resolution. Low-aspect-ratio nano/micropore analysis system with nanoscale thickness can provide a fast 2D scan to characterize the shape of analytical entities, which is not available with counting-only Coulter counters. When a charged target substance translocates through the nano/micropore under a DC electric field, ions in the nano/micropore channels are excluded, so a large current drop would be observed. The current pulse intensity depends on the size, charge, hydrophobicity, and morphology of target entity. Unlike biologically engineered channel used for gene sequencing (20), the significant advantages of the solid-state nano/micropores reported in this study are stable under extreme experimental conditions and can be integrated easily.

**Figure 1:**
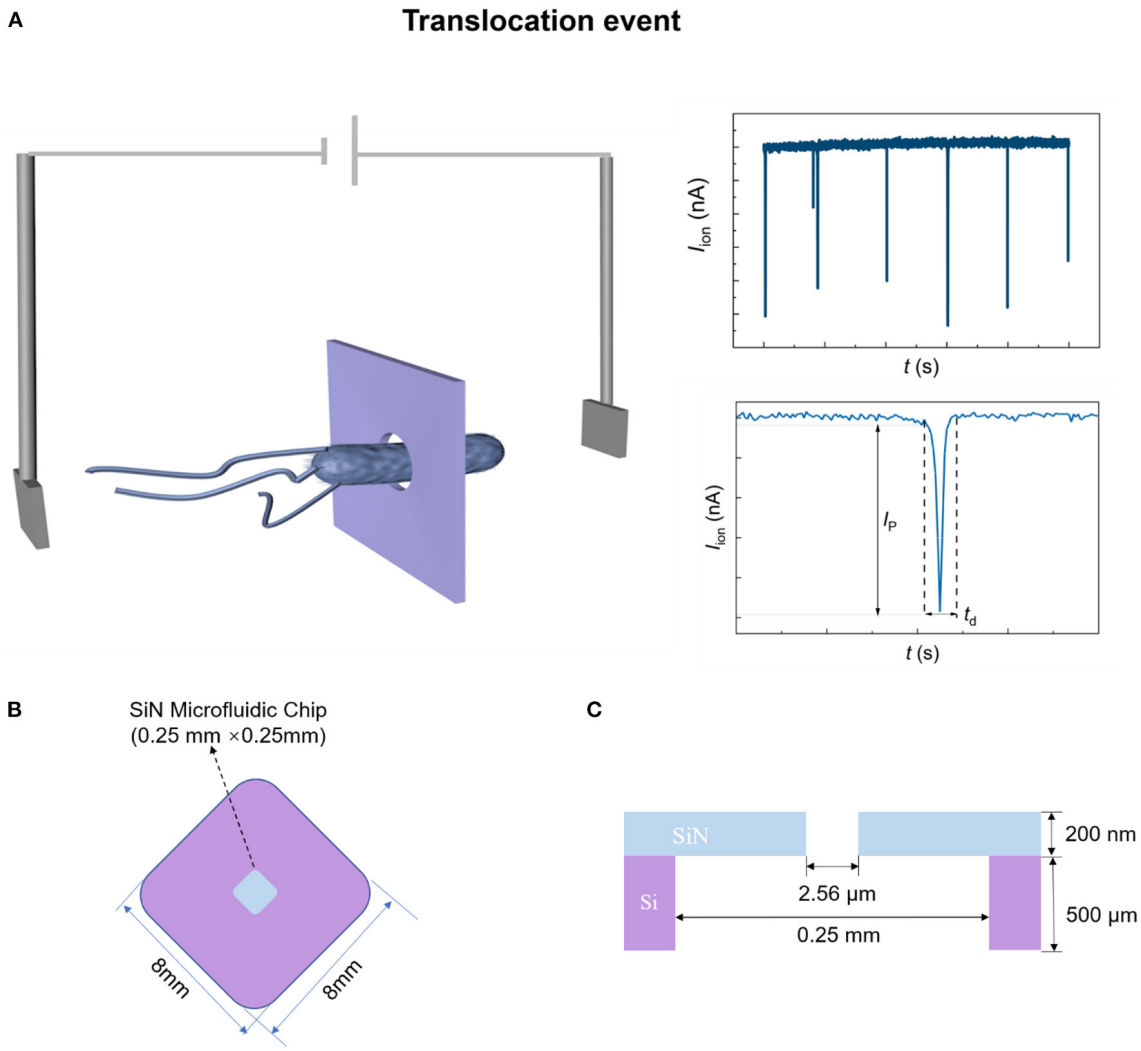
Schematic diagram of SiN chip sensing system. **(A)** Schematic diagram of bacterial translocation through a single micropore. **(B,C)** The top-view **(B)** and cross-sectional **(C)** structure diagram of the SiN chip.

In this Brief Research Report, we prepared a microfluidic chip with low-noise and low-aspect-ratio micropore for quick identifications of *Escherichia coli* (*E. coli*), *Salmonella enterica* (*S. enterica*), and *Listeria monocytogenes* (*L. monocytogenes*), which are commonly found in diverse food matrices. Since the bacterium is electrically charged, it can migrate through the micropore under an electric field, causing large current changes in a very short period of time. Each current pulse represents a bacterial translocation “event” (Figure 1A). Because of the differences in the physical parameters such as shape, charge, and mass of bacteria, different ionic current pulses are generated corresponding to the translocation of different bacteria. The sensing signal can be optimized by adjusting the pore size, voltage magnitude, sampling interval, etc. *E. coli, S. enterica*, and *L. monocytogenes* were identified according to the fine features of the resistive pulses. Due to convenience, rapidity, and accuracy, the label-free method, we report here has great potential for identification of the diverse foodborne microorganisms with the help of artificial intelligence sorting.

## Methods

### Chemicals and equipment

Electrochemical measurements were performed on a CHI-660e electrochemical workstation (CHI Instrument, Shanghai, China) with a microfluidic SiN pore in potassium chloride (Sinopharm Group, Shanghai, China) and 1 × PBS (Sangon, Shanghai, China). Luria–Bertani (LB) and Brain Heart Infusion (BHI) medium were purchased from the Beijing Land Bridge Technology Co. (Beijing, China). The bacteria used in the experiment were from the National Culture Collection Center (NCCC) (Beijing, China). Ultrapure water was used for the experiments. All the reagents and other chemicals used in this work were of analytical grade or better.

### Preparation and quantification of bacteria

*E. coli* (CICC 25922), *S. enterica* (CICC 10871), and *L. monocytogenes* (CICC 21635) were cultured in the shaking incubator for ~ 14 h at 37°C until the stationary phase in LB or BHI broth. After that, the obtained bacterial cells were centrifuged at 6,500 g for 5 min, washed twice, and resuspended in 0.1 × PBS for further use. Then bacteria were stained with 1 × SYBR Green and counted under an inverted fluorescence microscope.

### Fabrication of microfluidic chip

**Figure 2:**
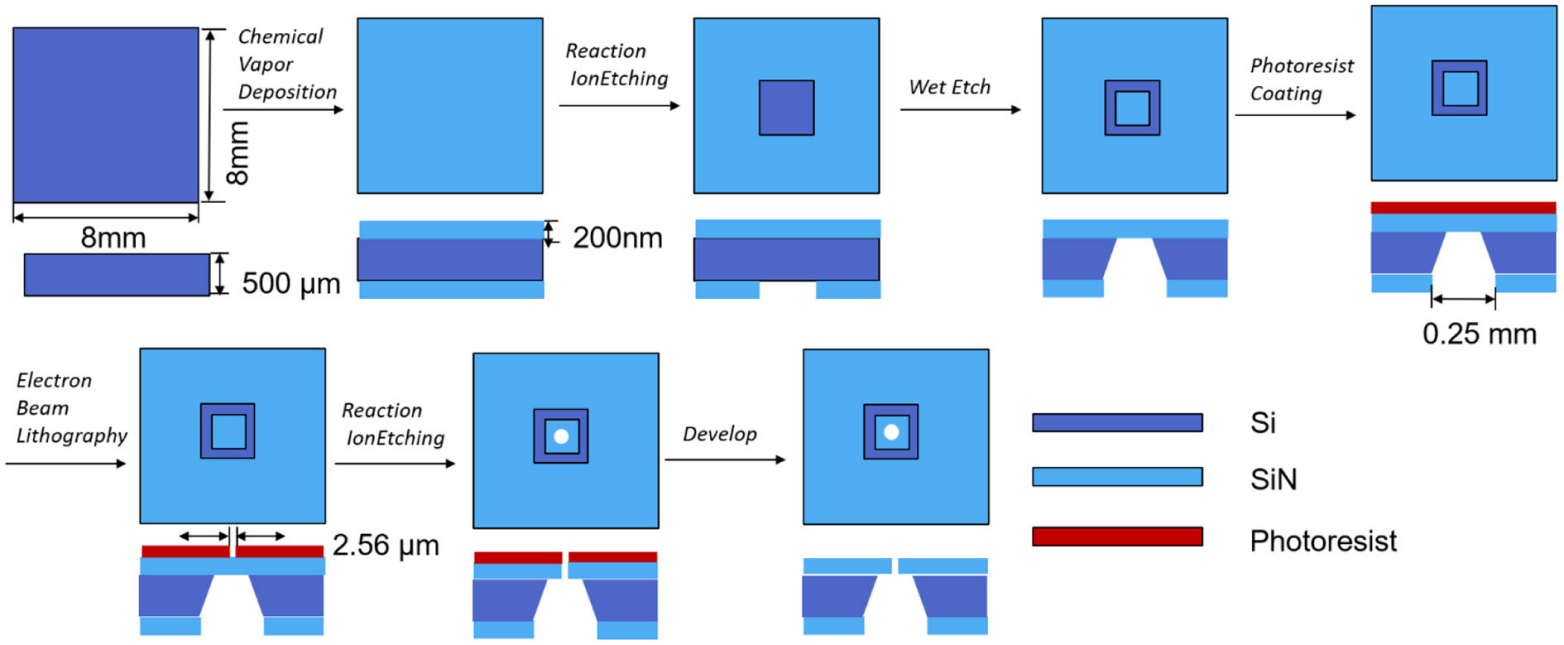
Fabrication process of SiN chip.

The microfluidic SiN chip was prepared according to a previously reported method (21). Briefly, a 500 μm thick Si wafer cut into 8 mm square chips was used as the substrate. On both the sides of the Si layer, there is 200 nm thick SiN formed by low-pressure chemical vapor deposition. The bottom side of the SiN was partially removed by reactive ion etching (RIE) through a mask with a square window of size 0.25 mm × 0.25 mm. Immerse the exposed Si in an aqueous KOH solution and heat to 80°C for wet etching, which resulted in the formation of a SiN film on the other side of the chip. A micropore with a diameter of 2.56 μm was prepared on the free-standing SiN film by electron beam lithography (Figure 2). After fabrication, the chip was baked at 100°C for 12 h with a piece of polydimethylsiloxane (PDMS) on the top. During heating, a thin PDMS layer would be coated on the SiN chip surface to reduce the capacitance of sensors (22), which will be discussed in detail in the Result section.

### Performance of micropore sensors for foodborne bacteria identification

The chip was mounted in between two halves of a conductivity cell. Both sides of the chip were filled with 0.1 × PBS. A pair of self-made Ag/AgCl electrodes were inserted into the cell. A constant voltage of 0.02V was applied across the chip and ionic current was measured over time by CHI 660e. For bacteria identification, a sample solution was added to one side of the cell. With the applied voltage, bacteria were electrophoretically driven through the pore and the temporal changes in ionic current across the pore were recorded. Peaks below the background baseline of the ionic current were defined as resistive pulses, the width and height of the pulse peaks were counted, frequency distribution histograms were plotted, and the accuracy of the current sensor for bacterial morphological classification was assessed.

## Results and discussion

### Structure of current sensing system

**Figure 3:**
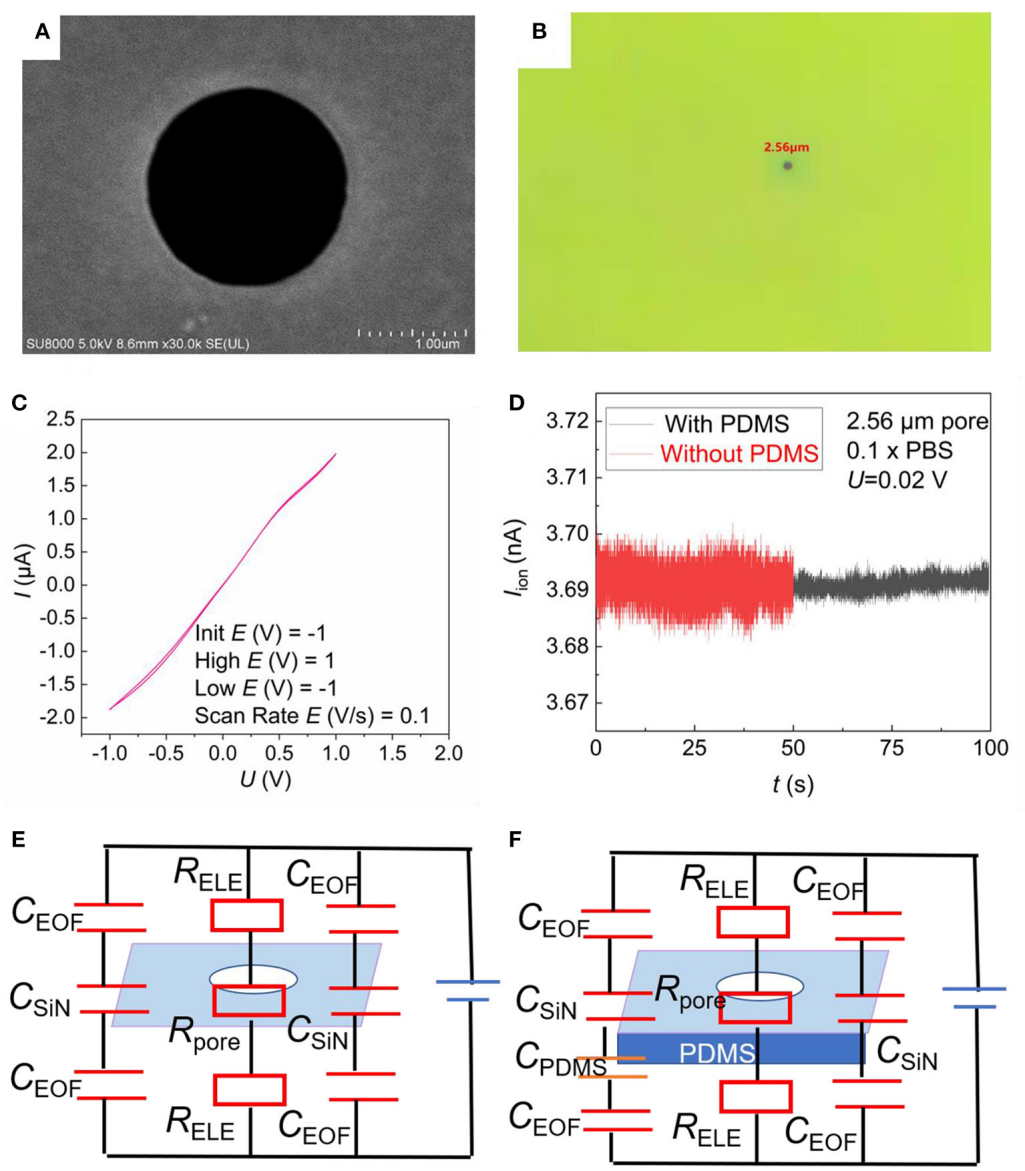
Characterization of SiN chips. **(A)** SEM photo image of a single pore on the SiN chip. **(B)** Optical photo image of single pore on the SiN chip. **(C)** Cyclic voltammograms of SiN chip recorded in the presence of 10 mM KCl as the electrolyte by a pair of Ag/AgCl electrodes. **(D)** *I*–*t* curve diagram of 2.56 μm SiN single pore chip with 0.1 × PBS buffer. **(E,F)** Equivalent circuits of the SiN-sensing system without PDMS **(E)** and with PDMS **(F)**.

Figures 1B,C show the schematic illustration of the top-view and cross-sectional structure of the microfluidic SiN chip, respectively. The SiN chip consists of a 200 nm-thick SiN layer supported by an 8 mm × 8 mm silicon wafer whose thickness is 500 μm, in the center of which there is a window of 0.25 mm × 0.25 mm. Scanning electron microscope (SEM) and *I–V* curves were used to characterize SiN chips. A single micropore with 2.56 μm in size can be seen in the SEM (Figure 3A) and optical photo (Figure 3B). The image is uniform in color, indicating that there are no voids and cracks on the free-standing SiN film. Therefore, SiN pores are the only channel for electrolyte transport across the membrane. Once the applied voltage and the electrolyte concentration were determined, the magnitude of the ionic current mainly depends on the resistance of the SiN micropore. Therefore, the pore diameter can also be characterized according to the magnitude of the ionic current. Concretely, the pore diameter of the SiN chip can be calculated by the following formula,


(1) $$\begin{array}{r} d=\sqrt{\frac{4l}{\left(\frac{U}{I}-R\right)k\pi }} \end{array}$$

Where *d* is the diameter of the micropore, *U* is the applied voltage, and *I* is the ionic current measured, *l* is the SiN membrane thickness, *R* is the resistance of the SiN window, *k* is the conductivity of the solution, which is 0.1413 S/m for 10 mM KCl at the experimental temperature. According to the current *I* measured in Figure 3C, we get *d*_1_ = 2.05 μm. This is basically consistent with the results measured in Figures 3A,B. The two lines of the cyclic voltammetry curve of the SiN chip basically coincide, and no peaks appear, indicating that there is no redox reaction, which reflects the stability of the SiN chip.

Figure 3D show the current of SiN pore measured in the blank PBS solution. The electric noise is significantly reduced after PDMS coating. To explain this phenomenon, we introduced the RC circuit model, as reported before (23). The whole-sensing system is a circuit composed of the resistance of the solution and SiN micropore, and the capacitance of the solid–liquid interface in parallel (Figures 3E,F). The total capacitance of this sensor (*C*_sensor_) can be calculated from Equation (2).


(2) $$\begin{array}{r} \frac{1}{C_{\text{sensor}}}=\frac{1}{C_{\text{EOF}}}+\frac{1}{C_{\text{SiN}}}+\frac{1}{C_{\text{PDMS}}} \end{array}$$

Here, *C*_EOF_ represents the electric double layer capacitance, *C*_SiN_ represents the capacitance of SiN, *C*_PDMS_ represents the capacitance of PDMS. When a thin PDMS is coated, the total capacitance of this sensor (*C*_sensor_) will be decreased. Therefore, the background noise in the *I*–*t* curve would be reduced and signal pulses will be more pronounced. Besides, the PDMS thermally deposited by high temperature is only a few nanometers thick according to previous research work (22), with no possibility to block our chip.

### Single bacteria sensing by microfluidic SiN chip.

**Figure 4:**
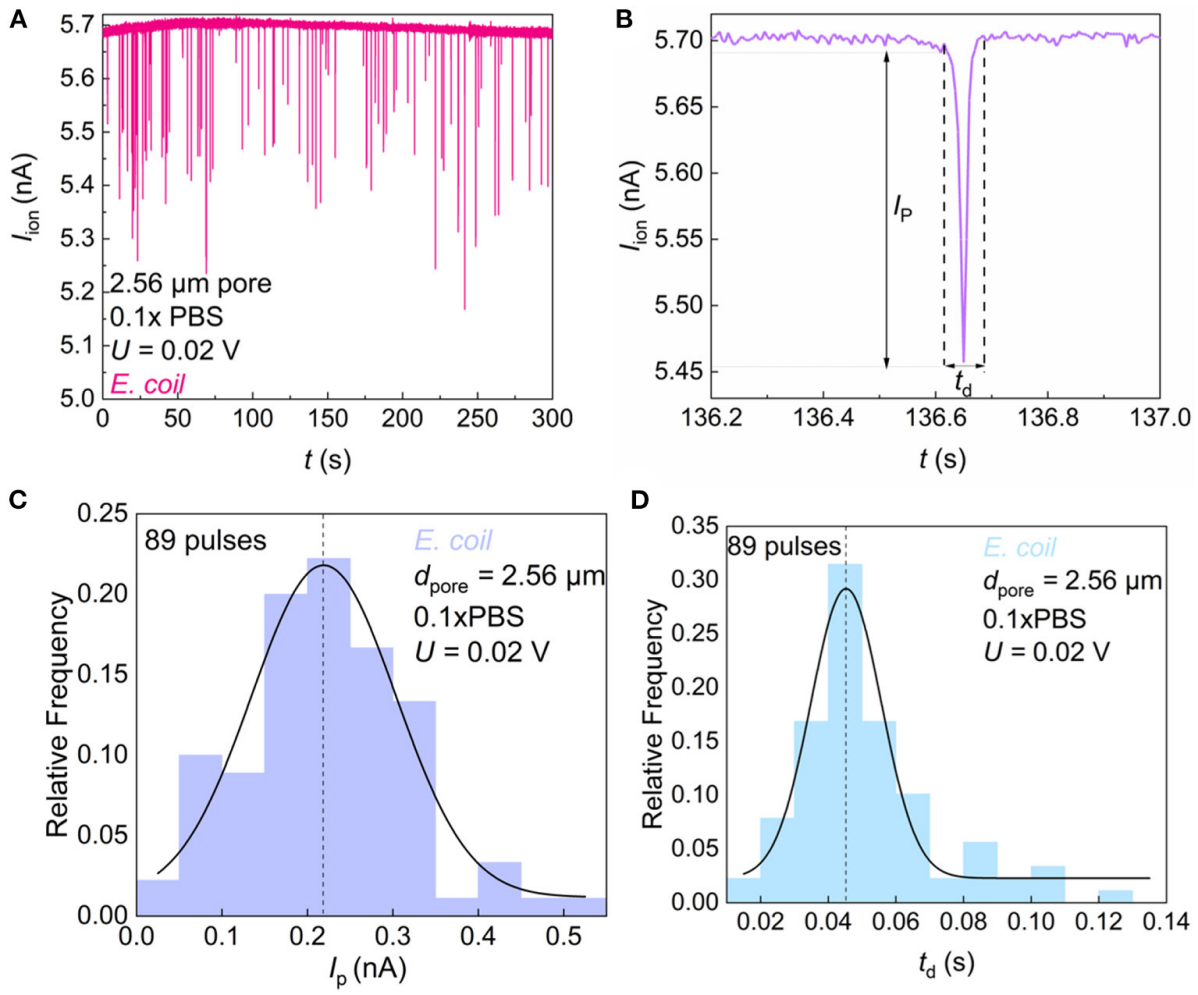
Sensing of single *E. coli* by microfluidic SiN chip. **(A)** *I*_ion_ vs. time (*t*) trace recorded in 0.1 × PBS containing *E. coli* with a micropore of diameter *d*_pore_ = 2.56 μm under *U* = 0.02 V. **(B)** Magnified views of resistive pulses in **(A)**. *I*_p_ and *t*_d_ denote the pulse height and width. **(C)** Frequency distribution of *E. coli* resistance pulse height. **(D)** Frequency distribution of *E. coli* resistance pulse width.

The ionic current signal of foodborne bacteria passing through the SiN pore was recorded. The electrical pulses were observed indicating that the bacteria were moving through the SiN pore (Figure 4A). When bacteria entered the micropore, the channel for electrolyte transport was blocked partially, thus the current decreased instantaneously. Here, we denote the pulse current exceeding the background current baseline as *I*_P_. Since each bacterium has a certain length, it takes a certain time for bacteria to pass through the pore. This length of time can be observed from the pulse peak shape, which we call *t*_d_ (Figure 4B). To better characterize the morphological characteristics of the bacterial species, the frequency distribution histogram of *I*_P_ (Figure 4C) and *t*_d_ (Figure 4D) were plotted. We count the pulse width and height in groups and fit the data with a Gaussian curve to obtain the mean and variance of bacterial resistance pulses. The smaller the variance, the smaller the difference between individual bacteria.

**Figure 5:**
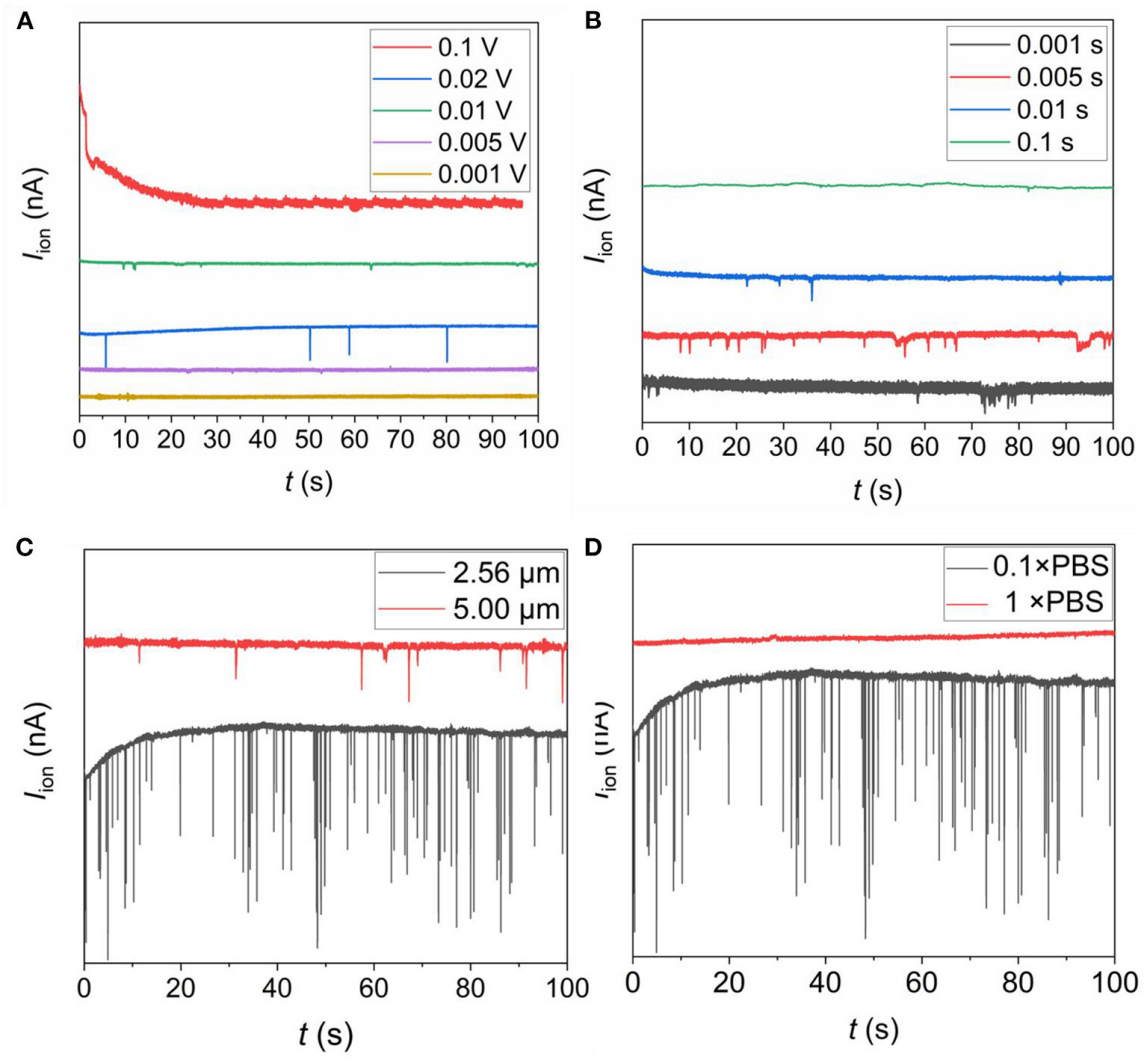
Optimization of SiN chip sensing system. **(A)** The *I*_ion_ traces of *Salmonella* passing through a 2.56 μm pore at different voltages with a sampling interval of 0.005 s in 0.1 × PBS buffer; **(B)** the *I*_ion_ traces of *Salmonella* passing through a 2.56 μm pore at a voltage of 0.01 V with different sampling intervals in 0.1 × PBS buffer; **(C)** the *I*_ion_ traces of *Salmonella* passing through a 2.56 μm and 5.00 μm pore at a voltage of 0.02 V with a sampling interval of 0.005 s in 0.1 × PBS buffer; **(D)** the *I*_ion_ traces of *Salmonella* passing through a 2.56 μm pore at a voltage of 0.02 V with a sampling interval of 0.005 s in 0.1 × PBS buffer and 1 × PBS buffer.

The size of the SiN single pore, the voltage applied across the chip, and the sampling interval of the ionic current and electrolyte concentration, all have an impact on the sensor performance. As shown in Figure 5A, resistive pulses were observed only when *U* = 0.01V and *U* = 0.02V, with *U* = 0.02V being the most obvious. When *U* = 0.1V, the ionic current curve was covered by the background noise, probably because of the fast passing of bacteria through SiN pore. The temporal resolution of our instrument was not able to capture such transient “events”. When *U* = 0.005V and *U* = 0.001V, the resistance pulse was not observed either. It may be because the bacterial movement was too slow under the voltage. As shown in Figure 5B, only when the sampling interval (hereinafter referred to as SP) was 0.01 or 0.005, the pulse peak was obvious. When SP was 0.1, the sampling interval was much longer than the time for the bacteria to pass through the micropore, and the transient “events” cannot be detected. When SP = 0.001, a large noise fluctuation was observed, which may mask the transient signal of bacterial translocate. The diameter of the SiN pore also has a great influence on the pulse signal. As shown in Figure 5C, the sensing signal of the 2.56 μm pore was significantly better than that of the 5.00 μm pore. This is because the 2.56 μm pore matches the size of most bacteria, and a large current change would be observed. As shown in Figure 5D, when bacterial sensing was performed in 1×PBS solution, no signal was observed. This may be attributed to the ionic conductance is too high because of the high-electrolyte concentration, so the voltage distributed across the solution is too small to drive the directional migration of charged bacteria. Therefore, we recorded the ionic current signal of different bacteria using a 2.56 μm SiN pore under the applied voltage of 0.02 V at a sampling rate of 200 Hz in a 0.1 × PBS buffer in the following experiments.

### Distinguishing different bacteria by the ionic current curve

**Figure 6:**
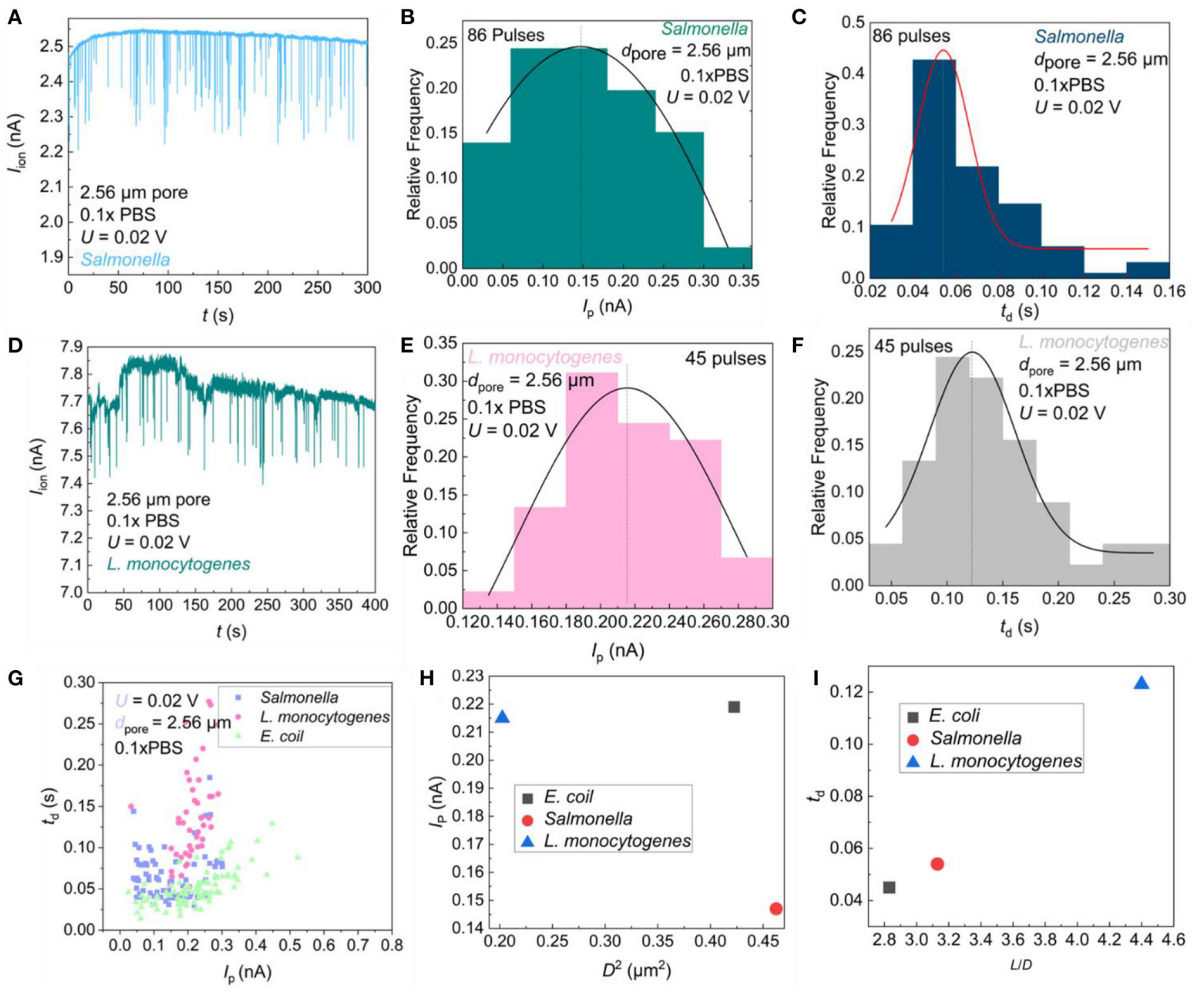
Statistical analysis of resistance pulses. *I*_ion_ vs. time (*t*) trace recorded in 0.1 × PBS containing *S. enterica* **(A)** and *L. monocytogenes* **(D)** with a single pore of diameter *d*_pore_ = 2.56 μm under *U* = 0.02 V; Frequency distribution of *S. enterica* **(B)** and *L. monocytogenes* **(E)** resistance pulse height; Frequency distribution of *S. enterica* **(C)** and *L. monocytogenes* **(F)** resistance pulse width; **(G)** The pulse height *I*_p_ vs. width *t*_d_ scatter plots of three bacterial; **(H)** Scatter plot of expected pulse height *I*_p_ vs. square of bacterial diameter for three bacteria; **(I)** The pulse width expected value *t*_d_ vs. bacterial aspect-ratio scatter plots of three bacteria.

The sensing signals for different bacteria were also measured as shown in Figure 6. When *S. enterica* and *L. monocytogenes* were present in the sample, pulse signals were also observed as shown in Figures 6A,D. It is obvious that different bacteria show different *I*_p_ and *t*_d_, which indicates the possibility for different bacteria sensing. In order to further distinguish the differences between different bacteria, the frequency distribution histogram of *I*_P_ and *t*_d_ were plotted. The *t*_d_ distribution of *S. enterica* (Figure 6C) was more concentrated than that of *E. coli* (Figure 4D) and *L. monocytogenes* (Figure 6F), while the *I*_p_ distribution of *E. coli* (Figure 4C) was more symmetrical than that of *S. enterica* (Figure 6B) and *L. monocytogenes* (Figure 6E). We found somewhat different Gaussian equations for different bacteria, which may be because of the differences in the size, charge, and physical morphology of bacteria. Figure 6G is a scatter plot of *I*_P_ vs. *t*_d_ for different bacteria. It is obvious that different bacteria were located at different positions in the figure, which indicates the possibility that we could use this method for bacterial classification.

The size of three pathogenic bacteria is shown in Table 1. The scatter plots of the signal height *I*_p_ as a function of the square of bacterial diameter (*D*^2^) and the pulse width *t*_d_ as a function of the aspect ratio of the bacteria (*L*/*D*) were constructed. As shown in Figure 6H, rod-shaped bacteria entering the micropore are not perpendicular to the pore, but have a certain inclination angle (24). The three types of bacteria have different shapes, so the angles of entering the pores are different. The diameter of *L. monocytogenes* is smaller than that of *E. coli*, so the volume of ions to be excluded from the pores is smaller than that of the *E. coli*, and the current drop is smaller than that of *E. coli*. The *S. enterica* enters almost at a vertical angle, so the volume of ions excluded is smaller than *E. coli* and *L. monocytogenes*, and a smaller current drop is obtained. The linear relationship of different bacteria with *t*_d_-*L/D* was observed in Figure 6I. This means that the more roundness of the bacteria, the less resistance it receives when passing through a single pore, and a faster translocation speed was found. In conclusion, the method reported in this study can well–reflect the physical shape differences of foodborne pathogens, which can be used for bacterial classification.

**Table 1:** Shape of three pathogenic bacteria and expected value of current pulse length and width (24).

**Foodborne bacteria**	**Length (μm)**	**Diameter (μm)**	**Aspect-ratio *(L/D)***	***I*_p_ (nA)**	***t*_d_ (s)**
*E.coli*	1.84	0.65	2.83	0.219	0.045
*S. enterica*	2.13	0.68	3.13	0.147	0.054
*L. monocytogenes*	1.98	0.45	4.40	0.215	0.123

## Conclusion

A simple microfluidic pore sensor was developed to classify three kinds of foodborne pathogenic bacteria commonly found in diverse food. Results demonstrated that the ion-blocking currents have adequate sensitivity to sense single-bacterium translocation, which allowed us to differentiate bacteria by analyzing the current pulses for their morphological characterization successfully. Factors such as bacterial size and single pore diameter, electrolyte concentration, applied voltage, and sampling interval that affect the signal were optimized in detail. This simple bacterial classification strategy has further practical applications because of its convenience and speed. But, the sensor still requires pre-processing of food samples to filter out the influence of large particles in complex matrices to prevent clogging of our microfluidic nano/micropores. In future work, we will focus on developing sensor chips that can be directly used for rapid detection of pathogenic bacteria in complex food matrices, and further improve their anti-interference and anti-fouling capabilities.

## Data availability statement

The original contributions presented in the study are included in the article/supplementary material, further inquiries can be directed to the corresponding authors.

## Author contributions

TY: investigation, formal analysis, visualization, and writing—original draft. ZSL: investigation, formal analysis, and visualization. RW: review and editing. LL: conceptualization and formal analysis. YQX: conceptualization and supervision. TD and XYL: critically revised and improved the manuscript. All authors contributed to the article and approved the submitted version.

## Funding

This work was supported by the National Key Research and Development Program of China (2019YFD1002300), the National Natural Science Foundation of China (22004107), the Zhejiang Provincial Natural Science Foundation of China (LR22C200002), and a project supported by Scientific Research Fund of Zhejiang University (XY2021017).

## Conflict of interest

The authors declare that the research was conducted in the absence of any commercial or financial relationships that could be construed as a potential conflict of interest.

## Publisher's note

All claims expressed in this article are solely those of the authors and do not necessarily represent those of their affiliated organizations, or those of the publisher, the editors and the reviewers. any product that may be evaluated in this article, or claim that may be made by its manufacturer, is not guaranteed or endorsed by the publisher.

## References

[1] Longo G Alonso-Sarduy L Rio LM Bizzini A Trampuz A Notz J. Rapid detection of bacterial resistance to antibiotics using AFM cantilevers as nanomechanical sensors. Nat Nanotechnol. (2013) 8:522–6. 10.1038/nnano.2013.120 23812189

[2] Lin X Fang M Yi C Jiang Y Zhang C Pan X. Functional hydrogel for fast, precise and inhibition-free point-of-care bacteria analysis in crude food samples. Biomaterials. (2022) 280:121278. 10.1016/j.biomaterials.2021.121278 34871876

[3] Yi C Luo Z Lu Y Belwal T Pan X Lin X. Nanoporous hydrogel for direct digital nucleic acid amplification in untreated complex matrices for single bacteria counting. Biosens Bioelectron. (2021) 184:113199. 10.1016/j.bios.2021.113199 33887613

[4] Logan NA Berkeley RCW. Identification of bacillus strains using the API system. Microbiology. (1984) 130:1871–82. 10.1099/00221287-130-7-1871 6432953

[5] Laure F Rouzioux C Veber F Jacomet C Courgnaud V Blanche S. Detection of HTV1 DNA in infants and children by means of the polymerase chain reaction. Lancet. (1988) 332:538–41. 10.1016/S0140-6736(88)92659-1 2900922

[6] Griffiths AJ Miller JH Lewontin RC Suzuki DT. An Introduction to Genetic Snalysis. 7th edition Whfreeman. (2000).

[7] de Macario EC Macario AJL. Monoclonal antibodies for bacterial identification and taxonomy: 1985 and beyond. Clin Lab Med. (1985) 5:531–44. 10.1016/S0272-2712(18)30858-8 3899480

[8] Carriço JA Pinto FR Simas C Nunes S Sousa NG Frazão N. Assessment of band-based similarity coefficients for automatic type and subtype classification of microbial isolates analyzed by pulsed-field gel electrophoresis. J Clin Microbiol. (2005) 43:5483–90. 10.1128/JCM.43.11.5483-5490.2005 16272474 PMC1287802

[9] Lv H Chen X. New insights into the mechanism of fluid mixing in the micromixer based on alternating current electric heating with film heaters. Int J Heat Mass Transf. (2021) 181:121902. 10.1016/j.ijheatmasstransfer.2021.121902

[10] Lv H Chen X Li X Ma Y Zhang D. Finding the optimal design of a Cantor fractal-based AC electric micromixer with film heating sheet by a three-objective optimization approach. Int Commun Heat Mass Transf. (2022) 131:105867. 10.1016/j.icheatmasstransfer.2021.105867

[11] Lv H Chen X Wang X Zeng X Ma Y. A novel study on a micromixer with Cantor fractal obstacle through grey relational analysis. Int J Heat Mass Transf. (2022) 183:122159. 10.1016/j.ijheatmasstransfer.2021.122159

[12] Lv H Chen X Zeng X. Optimization of micromixer with Cantor fractal baffle based on simulated annealing algorithm. Chaos Solitons Fractals. (2021) 148:111048. 10.1016/j.chaos.2021.111048

[13] Lin X Huang X Urmann K Xie X Hoffmann MR. Digital loop-mediated isothermal amplification on a commercial membrane. ACS Sensors. (2019) 4:242–9. 10.1021/acssensors.8b01419 30604619 PMC6350201

[14] Rothberg JM Hinz W Rearick TM Schultz J Mileski W Davey M. An integrated semiconductor device enabling non-optical genome sequencing. Nature. (2011) 475:348–52. 10.1038/nature10242 21776081

[15] Ouldali H Sarthak K Ensslen T Piguet F Manivet P Pelta J. Electrical recognition of the twenty proteinogenic amino acids using an aerolysin nanopore. Nat Biotechnol. (2020) 38:176. 10.1038/s41587-019-0345-2 31844293 PMC7008938

[16] Yang F Riedel R del Pino P Pelaz B Said AH Soliman M. Real-time, label-free monitoring of cell viability based on cell adhesion measurements with an atomic force microscope. J Nanobiotechnol. (2017) 15:23. 10.1186/s12951-017-0256-7 28330480 PMC5361698

[17] Ying Y-L Yu R-J Hu Y-X Gao R Long Y-T. Single antibody–antigen interactions monitored via transient ionic current recording using nanopore sensors. Chem Comm. (2017) 53:8620–3. 10.1039/C7CC03927A 28721409

[18] Arima A Harlisa IH Yoshida T Tsutsui M Tanaka M Yokota K. Identifying single viruses using biorecognition solid-state nanopores. J Am Chem Soc. (2018) 140:16834–41. 10.1021/jacs.8b10854 30475615

[19] Arima A Tsutsui M Harlisa IH Yoshida T Tanaka M Yokota K. Selective detections of single-viruses using solid-state nanopores. Sci Rep. (2018) 8:16305. 10.1038/s41598-018-34665-4 30390013 PMC6214978

[20] Van der Verren SE Van Gerven N Jonckheere W Hambley R Singh P Kilgour J. A dual-constriction biological nanopore resolves homonucleotide sequences with high fidelity. Nat Biotechnol. (2020) 38:1415–20. 10.1038/s41587-020-0570-8 32632300 PMC7610451

[21] Tsutsui M Yoshida T Yokota K Yasaki H Yasui T Arima A. Discriminating single-bacterial shape using low-aspect-ratio pores. Sci Rep. (2017) 7:17371. 10.1038/s41598-017-17443-6 29234023 PMC5727063

[22] Lin X Zhang B Yang Q Yan F Hua X Su B. Polydimethysiloxane modified silica nanochannel membrane for hydrophobicity-based molecular filtration and detection. Anal Chem. (2016) 88:7821–7. 10.1021/acs.analchem.6b01866 27414252

[23] Tsutsui M Yokota K Arima A Tonomura W Taniguchi M Washio T. Temporal response of ionic current blockade in solid-state nanopores. ACS Appl Mater Interfaces. (2018) 10:34751–7. 10.1021/acsami.8b11819 30204405

[24] Hattori S Sekido R Leong IW Tsutsui M Arima A Tanaka M. Machine learning-driven electronic identifications of single pathogenic bacteria. Sci Rep. (2020) 10:15525. 10.1038/s41598-020-72508-3 32968098 PMC7512020

